# On the relationship between blend state and dispersibility of adhesive mixtures containing active pharmaceutical ingredients

**DOI:** 10.1016/j.ijpx.2020.100069

**Published:** 2020-12-24

**Authors:** Jonas Rudén, Göran Frenning, Tobias Bramer, Kyrre Thalberg, Göran Alderborn

**Affiliations:** a Department of Pharmaceutical Biosciences and the Swedish Drug Delivery Forum (SDDF), Uppsala University, Husargatan 3, Box 580, SE-751 23 Uppsala, Sweden; b Inhalation Product Development, Pharmaceutical Technology & Development, Operations, AstraZeneca, Gothenburg, Sweden; c Emmace Consulting AB, Medicon Village, Lund, Sweden

**Keywords:** Adhesive mixture, Dry powder inhaler, Powder mechanics, Blend state, Fine particle fraction, Powder dispersibility

## Abstract

The objectives of this investigation were to study the evolution in blend state of adhesive mixtures containing the active pharmaceutical ingredients (APIs) salbutamol, budesonide and AZD5423 and to study the relationship between blend state and dispersibility of the mixtures, as assessed by the fine particle fraction (FPF). A series of adhesive mixtures of varied fines concentration were prepared for each API using the same type of carrier. Based on visual examination and powder mechanics, blend states were identified and summarized as blend state maps for each API. The dispersibility of the mixtures was studied using a Fast Screening Impactor (FSI) equipped with a ScreenHaler. The evolution in blend state differed between the APIs in terms of the width of the blend states. The structure of the adhesion layer also differed between the APIs, from relatively uniform to a heterogeneous layer with small agglomerates dispersed on the carrier surface. All three APIs expressed a similar type of bended relationship between FPF and fines concentration. However, the initial rate of increase and the fines concentration of the plateau differed between the APIs. The adhesive mixtures of all APIs followed the three main states in terms of structural evolution and the overall shape of the FPF-fines concentration profiles could be explained by the evolution in blend state. It is proposed that the structure of the adhesion layer is an important factor explaining the differences in blend state - blend dispersibility relationships between the APIs.

## Graphical abstract

**Figure:**
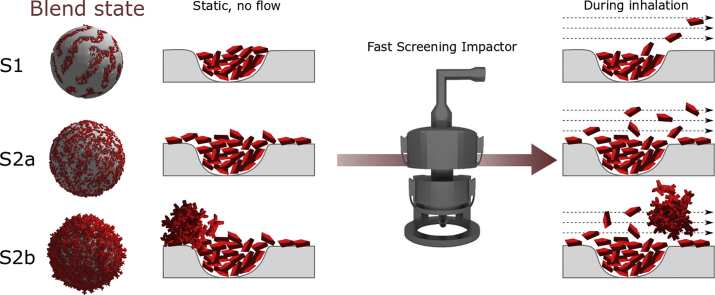

## 1 Introduction

Inhalation powders are often formulated as adhesive mixtures (de Boer et al. 2017; Grasmeijer et al. 2015) which consist of a special type of agglomerate formed from micron-sized drug particles mixed with larger, inert, carrier particles. The diameter of the drug micro-particles is typically within 1–5 μm while the carrier particles are of a diameter well above the inhalable range, i.e. about 60–150 μm (Pilcer and Amighi 2010). The role of the carrier material is to ensure homogeneity, powder flow and aerosolization performance during manufacturing and use (Thalberg et al. 2004). These types of formulations have been extensively investigated and discussed in the literature either by experiments, e.g. (De Boer et al. 2005; Dickhoff et al. 2003), or by simulation, e.g. (Nguyen et al. 2015; Sarangi et al. 2019) and the structural evolution of an adhesive mixture with increasing proportions of micro-particles (increased drug loading) have been described in the literature in terms of a consecutive series of blend structures (Hertel et al. 2018; Young et al. 2011). As a means to describe this structural evolution in a concentrated yet representative way we have proposed (Rudén et al. 2018; Rudén et al. 2019) a description referred to as a blend state model. The term blend state refers to the spatial distribution of carrier and micro-particles in an adhesive mixture and a blend state map shows the evolution in blend state versus the theoretical surface coverage ratio, or alternatively the proportion of fines, of a mixture of a certain combination of carrier and micro-particle. Hence, the blend state model can be used to map different combinations of micro-particles and carriers (i.e. blend state map).

Based on experiments using one type of lactose carrier and one type of lactose micro-particle (Rudén et al. 2018), we have proposed that a blend state model can consist of up to four regions (denoted S1, S2a,S 2b and S3) and in-between each state a transition step or transition interval exists. State 1 is associated with the deposition of micro-particles in surface cavities of the carrier, also referred to as active sites in the literature (Yeung et al. 2018). In state 2, micro-particles will adhere to the enveloped (outer) surface of the carriers and an enveloped adhesion layer is formed, which will gradually increase in thickness. State 2 can be sub-divided into state S2a and S2b dependent on the appearance and dynamics of the adhesion layer. Finally, in state 3, free self-agglomerates of micro-particles are formed. The determination of blend states and transition steps was done by using experimental data on the packing and flow of the mixtures and by close inspection of the mixtures by microscopy. In a follow-up study (Rudén et al. 2019), we derived blend state maps for five different carriers and one type of micro-particle. Considerable differences in blend state maps were obtained dependent on the carrier size and morphology. This includes the width of region 2, which relates to an upper limit of drug loading of this type of inhalation powder.

Young et al. (2011) studied how the drug loading of adhesive mixtures affected their aerosol performance and reported that the performance was dependent on the drug loading proposed to be mediated by the blend structure. Thus, knowledge of the blend state may be critical in order to understand the aerosolization performance of adhesive mixture during inhalation. Important in this context may be the sequential relationships between blend state, mixture dispersibility and aerosolization performance. The dispersibility can be defined as the propensity of the adhesive units to disperse or aerosolize while subjected to an air stream. The dispersion involves the detachment of fine particles either as single particles or as agglomerates from the carrier surfaces. In the case of agglomerates, they need to be further deagglomerated into primary particles or small particle clusters in order to be inhaled. Since different fine particles will need different forces to detach and deagglomerate, the degree of dispersion is dependent on the dispersion intensity or energy, typically the flow dynamics of the air (Crowder et al. 2002). In practice, the dispersibility is often assessed by the fine particle fraction of the dose under defined air flow conditions (Stegemann et al. 2013).

In our earlier studies (Rudén et al. 2018; Rudén et al. 2019), both the carriers and the micro-particles used were α-lactose monohydrate. The rationale behind this choice was to have a constant adhesion strength of the variety of particle-particle interactions in the mixture. The relative strength of the particle-particle interactions of an adhesive mixture that consists of particles of different materials, hence showing different surface properties, is sometimes denoted the cohesion-adhesion balance of the particle-particle interactions. It is reported (Jones and Buckton 2016) that the cohesion-adhesion balance is critical for the performance of an inhalation powder. We therefore now intend to study how the evolution in blend state varies between different types of micro-particles. To that end, mixtures of one type of carrier and three APIs (budesonide, salbutamol and AZD5423) were studied and the question how the drug will affect the blend state map and the powder dispersibility was addressed. The aims of this study were thus firstly; to study the effect of fines content on the blend state, as described by blend state maps, of adhesive powder mixtures consisting of one type of carrier and three different drug micro-particles and secondly; to study the dispersibility of these adhesive mixtures and, finally; to examine the blend state-blend dispersibility relationships.

## 2 Materials and methods

### 2.1 Materials

An α-lactose monohydrate carrier, Lactopress SD (DFE Pharma, The Netherlands), and three micronized active pharmaceutical ingredients (API), i.e. Budesonide, Salbutamol and AZD5423 (PubChem 2020) (all obtained from AstraZeneca Gothenburg, Sweden), were used in this study. The apparent particle density of the materials was determined by Helium pycnometry (AccuPyc 1330, Micromeritics Instruments, Norcross, USA) using a 10 ml steel cylinder filled up to approximately 30% of its volume with the sample material. The reported values in Table 1 are the average apparent particle density from 5 subsequent measurements with standard deviation.

**Table 1:** Particle and powder characteristics of fines and carrier. Reported values are the mean and standard deviation (*n* = 3) for all except BET (*n* = 2).

Material	Particle density (g/cm^3^)	Specific surface area, perm (cm^2^/g)	Specific surface area, BET (cm^2^/g)	Particle size (D_50_, μm)	Span	Bulk density (g/cm^3^)	Porosity (bulk)
Lactopress SD	1.54 (0.00)	778 (10.3)	1905	110 (0.55)	1.21	0.617 (0.00)	0.60
Budesonide	1.28 (0.00)	50,636 (2093)	56,045	1.62 (0.02)	2.06	0.159 (0.00)	0.88
Salbutamol	1.34 (0.00)	59,323 (280)	47,323	1.87 (0.02)	2.03	0.123 (0.00)	0.91
AZD5423	1.38 (0.00)	36,572 (809)	161,465	1.80 (0.01)	2.15	0.181 (0.00)	0.87

For the UPLC analysis (see 2.4, 2.5), LC grade methanol and acetonitrile were used (Merck KGaA, Darmstadt, Germany). MilliQ water was produced using a Purelab Flex (ELGA LabWater, United Kingdom) operated at 18.2 Ω. The compounds used as internal standards (fluocinolone acetonide and 4-propyl hydroxy benzoate) as well as the orthophosphoric acid, sodium dihydrogen phosphate and trifluoroacetic acid (TFA) were purchased from Merck/Sigma Aldrich (Merck KGaA, Darmstadt, Germany).

### 2.2 Particle characterization

#### 2.2.1 Particle size

The particle size distribution of the materials was determined by laser diffractometry using a Sympatec HELOS laser diffraction instrument (Sympatec GmbH, Clausthal-Zellerfield, Germany). To disperse the materials before measurement, a dispersion pressure of 4 Bar was used. The carrier was analysed using the R5 lens (0.5 to 875 μm) and the APIs was analysed with the R1 lens (0.3 to 80 μm). The particle diameter (D) was derived using Fraunhofer theory and D_10_, D_50_, D_90_ were determined from a cumulative volume distribution. The span of the distribution was calculated as reported earlier (Rudén et al. 2018). The reported values in Table 1 are the mean and standard deviation from three measurements.

#### 2.2.2 Particle specific surface area

The specific (external) surface area for the fine particles and the carrier was measured by air permeametry as described in earlier work (Alderborn et al. 1985; Rudén et al. 2018). In short, a Blaine permeameter was used for the APIs and a steady-state permeameter was used for the carrier. The specific surface area was calculated by the Kozeny-Carman equation with (API) or without (carrier) slip-flow correction. The samples used during permeability measurements had an average porosity of 54–59%. The reported values are the mean and standard deviation from three measurements (Table 1).

The BET specific surface area of fines and carrier particles was determined by gas adsorption (TriStar III 3020, Micromeritics Instruments, Norcross, USA) using nitrogen as adsorbent. The surface area was calculated using the BET equation from six data points within a relative pressure range of 0.05 and 0.35 (Brunauer et al. 1938). Prior to the measurement, the samples were degassed with nitrogen for two hours at 40 °C using a SmartPrep device (Micromeritics Instruments, Norcross, USA). The reported values are the mean from two measurements.

#### 2.2.3 Particle and mixture morphology

Scanning electron microscope images were taken of all powders and mixtures using a Hitachi TM3030Plus (Hitachi, Tokyo, Japan) for the mixtures and a Zeiss 1530 (Carl Zeiss GmBH, Oberkochen, Germany) for the APIs. A few scoop sampled particles were sprinkled over a carbon tape followed by gentle tapping to remove excess powder. The samples were then gold coated using a Cressington 108 auto sputter-coater (Cressington Scientific, Watford, UK). The Hitachi TM3030Plus was operated at an acceleration voltage of 5 kV with images captured at 100-500× magnification using a mixed BSE/SE detector. The Zeiss 1530 was operated at an acceleration voltage of 2.5 kV with images captured at 5-10kx magnification using an InLens detector.

To estimate the point of self-agglomeration, light microscope images were taken of mixtures when larger self-agglomerates started to visually appear in the blends. The images were taken using a Zeiss SteREO Discovery.V8 (Carl Zeiss GmBH, Oberkochen, Germany) at 1× magnification.

### 2.3 Preparation of adhesive mixtures

A series of adhesive mixtures were prepared for each API using a Turbula T2F mixer (Willy A. Bachofen AG, Switzerland). Prior to mixing, the fines were sieved through a 710 μm sieve. Thereafter, a 200 ml glass vessel was filled with 40–60 g of carrier powder and API powder, giving approximately 50% of the vessel volume filled with powder, and mixed for 1 h at 46 rpm. Different weights of fine particles were used in the mixtures dependent on the weight proportion of fines (Table 2). The proportions of powder used in the mixtures corresponded to a series of surface coverage ratio (SCR) up to a SCR of 2, using the same approach to calculate the SCR as described earlier (Rudén et al. 2018). For budesonide, two additional mixtures were prepared at SCR of 3 and 4 to reach the SCR at which self-agglomeration of the API was visually observed.

**Table 2:** Proportions of fines in all mixtures.

SCR	Budesonide (%)	Salbutamol (%)	AZD5423 (%)
0.25	1.21	1.03	1.67
0.5	2.41	2.06	3.34
0.75	3.62	3.09	5.01
1	4.83	4.12	6.68
1.5	7.24	6.18	10.0
2	9.65	8.24	13.4
3	14.5		
4	19.3		

### 2.4 Assessment of mixture homogeneity

To assess the mixture homogeneity, mixture samples were drawn from some selected adhesive mixtures and the content of API in the samples subsequently determined by liquid chromatography. A Waters Acquity UPLC system (Waters Corp, Milford, USA) equipped with a C18 BEH 1.7 μm 2.1 × 50 mm column and a photo diode array (PDA) detector was used to analyse the API content. Three mixtures per API, representing different blend states and thus API content, were selected for the analysis. A sample weight of about 15–20 mg represented an adequate scale of scrutiny for the homogeneity analysis. Initial powder sampling trials were done by using an end cup sampling thief probe. It was however concluded that self-agglomerates of API in the mixture were pushed away by the thief probe and the device could not be used to draw representative samples at the chosen scale of scrutiny. Thus, simple scoop sampling was used by which samples could be drawn which also included self-agglomerates. For each of the selected mixtures, 10 samples of 15–20 mg were drawn from randomly selected places of a bed of the mixture held in the same container as used during powder mixing. Similar positions within the mixture bed from which the samples were drawn were used for all mixtures. The samples were weighed and then transferred into a vial in which the samples were dissolved in a solution with or without an internal standard depending on the API (see section 2.5). The concentration of API of each sample was calculated using calibration curves in the concentration range. The concentration of API was then normalized to a constant sample weight of 15.0 mg and the variation in API concentration between the samples, expressed as the relative standard deviation, was used as an indication of blend homogeneity.

### 2.5 Assessment of mixture dispersibility

To assess mixture dispersibility, a Fast Screening Impactor (FSI) (Copley Scientific, UK) and a low resistance ScreenHaler device (Thalberg et al. 2016) were used. The volumetric rate of airflow was set to 60 ± 0.3 L/min and the suction time to 4 s (4 L total suction volume) using a Triggbox model III (FIA AB, Lund, Sweden).

Prior to testing, the inhaler device was manually filled with a dose varying between 15.0 and 17.5 mg for each single dispersibility test. For the mixtures with the lowest concentrations of API, 2–3 actuations were used in order to reach quantifiable amounts in the chemical analysis, using the same UPLC instrument as mentioned in 2.4. The amount of API was determined at the three stages of the impactor, i.e. throat, pre-separator and filter. The samples were collected from each of these stages by first adding an internal standard solution or a specific amount of solvent (20 ml). Following this, the throat and pre-separator were set to shake using a Sample Preparation Unit (Copley Scientific, UK) for 20 mins, while the filter was transferred to a petri-dish and set to shake on a shaking table for the same duration. After the sample preparation was completed, 0.5 ml from each stage was transferred into separate LC-vials for analysis. In the case of budesonide, 0.8 ml of phosphate buffer solution (pH 3.2) was added to the LC vials before the analysis.

For salbutamol and budesonide, the mobile phases were water and acetonitrile, both with 0.03% TFA. For AZD5423, the mobile phases were water with 0.06% orthophosphoric acid and pure methanol. The salbutamol sample preparation involved only the addition of water and hence no internal standard was used. For budesonide and AZD5423, the solutions included ethanol and thus internal standards were used to avoid the influence of significant evaporation. The internal standards used were fluocinolone acetonide for budesonide and 4-propyl hydroxy benzoate for AZD5423 (concentrations approx. 20 mg/L). The UV wavelength was set to 219 nm for salbutamol and 254 nm for both budesonide and AZD5423.

The amount of API in each stage was quantified from the response factors or area under the curve (AUC) using calibration curves in the concentration range. The fine particle fraction (FPF, %) was then calculated as follows (Eq. 1),

(1) $$\mathrm{FPF}=\frac{\mathrm{Filter stage}}{\mathrm{ED}}\times 100$$

where the filter stage represents the amount of drug (μg) deposited on the filter (aerodynamic cut-off of 5 μm) and ED (emitted dose) is the sum of drug (μg) on all three stages. The reported values are the mean and standard deviation from three measurements.

### 2.6 Assessment of powder mechanics

#### 2.6.1 Conditioning of powders

All powders were pre-conditioned by storage in a climate-controlled room for at least one day at a temperature of 21–24 °C and a relative humidity (RH) of 30–34% before any experiments were carried out. All powder mechanical experiments were performed in the same climate conditions. The dispersibility experiments were performed at ambient conditions, i.e. room temperature and ~ 30–40% RH.

#### 2.6.2 Unsettled bulk density

The unsettled bulk density was determined as previously described (Rudén et al. 2018; Rudén et al. 2019) using a device manufactured by AstraZeneca Gothenburg (Mölndal, Sweden) consisting of a steel sample container of 20.05 ml. In short, the measurement involved the use of an inner cylinder, placed inside the sample container, which was slightly higher than the container. The cylinder was filled with powder and thereafter lifted so that the powder flowed into the sample container. The reported values are the mean and standard deviation from three measurements.

#### 2.6.3 Compressibility

The compressibility of powders, i.e. adhesive mixtures and only API powders, was determined using a FT4 Powder Rheometer as described earlier (Rudén et al. 2018). In short, the method involved the filling of a 10 ml sample container, equipped with a perforated metal base plate, with powder whereafter the powder was compressed with a ventilated steel piston using a series of normal stresses ranging from 1 to 30 kPa. During the compression, an airflow of 2 mm/s was maintained. The height of the powder bed held in the sample container of the rheometer was assessed and as a measure of powder compressibility, the Hausner ratio was calculated, i.e. the ratio between the height of the sample container and the height of the powder bed after compression. The reported Hausner ratios are the mean of three measurements.

## 3 Results

### 3.1 Material characteristics

#### 3.1.1 Particle size distribution and apparent density

The apparent particle density of the materials were in the range of 1.28–1.54 g/cm^3^ (Table 1). The carrier, Lactopress SD, had a median particle diameter of 110 μm with a span of 1.21. The three APIs were of similar median particle diameter, varying between 1.62 and 1.87 μm, and of a similar spread in particle diameter with a span of about 2.

#### 3.1.2 Particle morphology

**Fig. 1:**
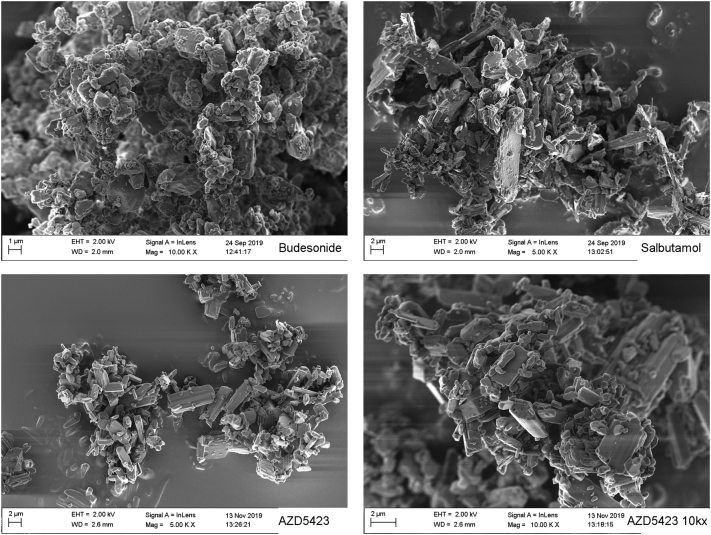
Scanning electron microscope images of all fines.

SEM images of all APIs are presented in Fig. 1. The Lactopress carrier, as described in a previous paper (Rudén et al. 2018), was a highly corrugated spray-dried lactose with relatively regular, nearly spherical geometrical shape. The primary micro-particles of budesonide seemed relatively regular in geometrical shape and the larger particles had smooth surfaces. For budesonide, some particles appeared to be significantly smaller than the median particle diameter, which could be the reason why budesonide had the lowest D_50_. Regarding the salbutamol and AZD5423, the particles were irregular in geometrical shape with elongated rod shaped particles. Some of the particles appeared to be well above the median particle diameter of around 1.8 μm.

#### 3.1.3 Specific surface area

The specific surface area of the materials was assessed by two principally different techniques (Table 1), i.e. permeametry surface area and gas adsorption BET surface area. Budesonide and salbutamol expressed the highest permeametry surface areas, which also were similar to their BET surface areas. AZD5423 on the other hand, had a permeametry surface area considerably lower than the BET area. SEM image of AZD5423 at a higher magnification (Fig. 1) showed that the particles had open nano-sized pores.

#### 3.1.4 Powder mechanics of fine materials

The unsettled bulk density and the bulk porosity of the fine materials are presented in Table 1. Salbutamol powder had the lowest unsettled bulk density and the highest bulk porosity among the fine powders although it had the highest measured median particle size. The generally low bulk densities and high bulk porosities mean that the powders packed very loosely due to the small particle diameter. The salbutamol powder had a higher porosity than budesonide and AZD5423 powders, which reflects differences in primary particle shape. The AZD5423 powder expressed the highest unsettled bulk density, which is at least partly explained by a higher particle density but may also be due to a higher degree of agglomerates present in the powder, which hence can pack more densely under unsettled conditions.

**Fig. 2:**
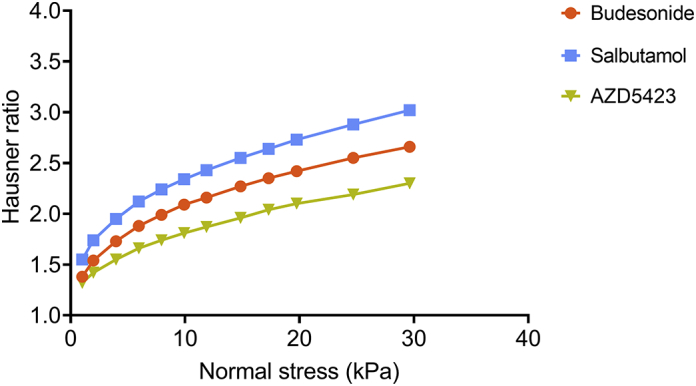
Effect of applied normal stress on the Hausner ratio of powders of the fines. Average values (*n* = 3) with standard deviation (some too low to be visible).

In Fig. 2, the Hausner ratio as a function of the applied normal stress is presented for all fine materials. Salbutamol was the most compressible material, probably due to the low unsettled bulk density, followed by budesonide.

### 3.2 Mixture structure and homogeneity

#### 3.2.1 Mixture structure

**Fig. 3:**
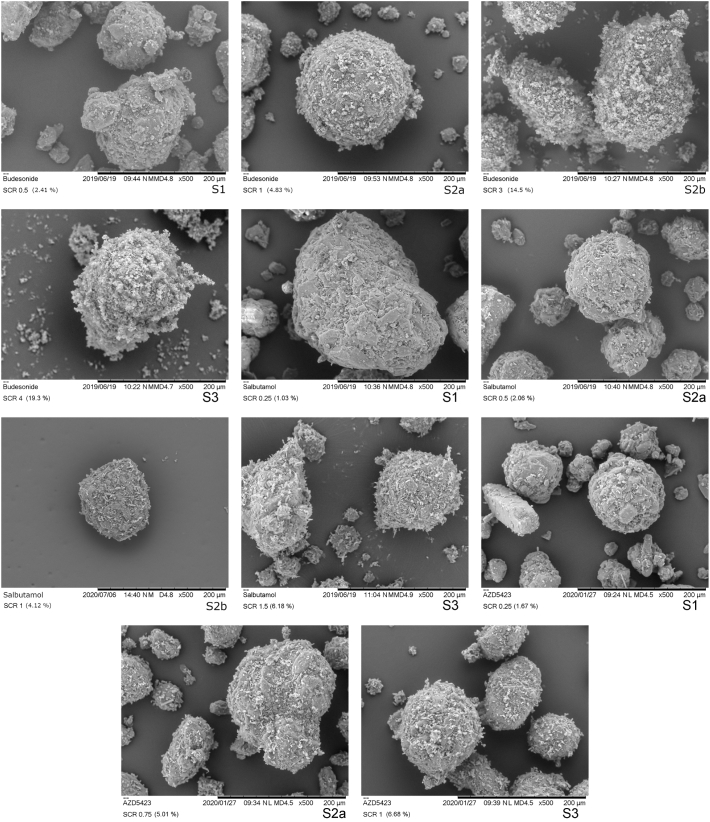
Scanning electron microscope images of adhesive mixtures representing the observed blend states.

**Fig. 4:**
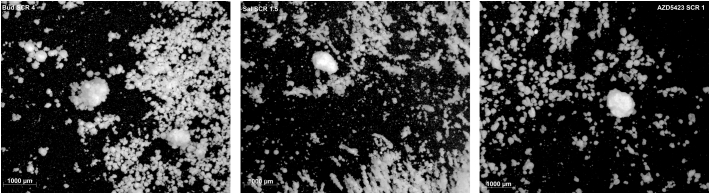
Light microscope images of adhesive mixtures at the beginning of stage 3, illustrating the size and morphology of self-agglomerates.

The physical structure of carrier-API mixtures was studied by SEM imaging (Fig. 3) and light microscopy (Fig. 4). The SEM images presented here represents one mixture for each blend state of the mixture (see below for API concentrations for the respective blend state). For blend state 1 and 2, the selected mixtures are the mixture with the highest fines concentration within each blend state, i.e. the concentration before the mixture transits into the next blend state. For blend state 3, the selected mixtures are the lowest concentration of fines in this state.

Typically, the S1 state is characterized by the adhesion of fines to cavities or open pores of the carrier (Rudén et al. 2018). For adhesive mixtures of low proportions of API, the fines were predominantly localised in surface cavities and only a limited number of particles were attached to the enveloped surface of the carrier particles. Thus, blend state 1 was expressed for all APIs used but the concentration of fines differed between the fine materials at the upper limit of blend state 1.

With increased proportion of fines in the mixture, an increased amount of fine particles began to attach to the outer surface of the carrier, i.e. S2 was formed for all APIs. Both single fine particles and small self-agglomerates of fines were attached to the carrier in S2. With further increase in fines concentration, a larger fraction of the enveloped carrier surface became covered with fines and the number of attached self-agglomerates increased, categorized as a transition from S2a to S2b. Eventually, free self-agglomerates, i.e. self-agglomerates that are not attached to the carrier surface, were formed and thus the S3 blend state was reached for all APIs (Fig. 4). The formation of free self-agglomerates occurred at varying SCR:s or fines concentration depending on the API. Budesonide appeared to be the API that required the highest concentration of fines (SCR 4 about 19.3%) before self-agglomerates started to appear. AZD5423 started to self-agglomerate at a SCR of about 1, corresponding to similar fines concentrations as the point of self-agglomeration for salbutamol (SCR 1.5). The size and shape of the self-agglomerates differed between the APIs and the size of the self-agglomerates, as observed by light microscopy, increased in the following order (approximate fines concentration within brackets): Budesonide (19.3%) < AZD5423 (6.68%) < salbutamol (6.2%).

#### 3.2.2 Homogeneity

The homogeneity or local variation in API content, as assessed by the relative standard deviation (RSD) of the variation in API concentration between samples of 15–20 mg, are presented in Table 3. All tested mixtures had a relative standard deviation below 5% except for the mixture of the highest concentration of AZD5423, which gave a RSD of 9.19%. In Table 3, the measured amount and the theoretical amount of API in the mixtures are also presented. The measured amount of API was lower for all mixtures except for salbutamol at SCR 1 and AZD5423 at SCR 0.25.

**Table 3:** Measured amount and theoretical amount of API in mixtures. Average values (n = 10) with relative standard deviation. Average values (*n* = 10) with relative standard deviation.

SCR	Budesonide			Salbutamol			AZD5423		
	Average (μg)	Rel StD (%)	Theoretical (μg)	Average (μg)	Rel StD (%)	Theoretical (μg)	Average (μg)	Rel StD (%)	Theoretical (μg)
0.25	170	1.94	181	139	3.69	154	255	1.51	251
1	646	1.93	724	676	3.36	618	972	4.22	1002
2	1281	1.21	1448	1100	2.65	1236	1802	9.19	2005
4	2392	1.41	2896						

### 3.3 Powder mechanics of adhesive mixtures

**Fig. 5:**
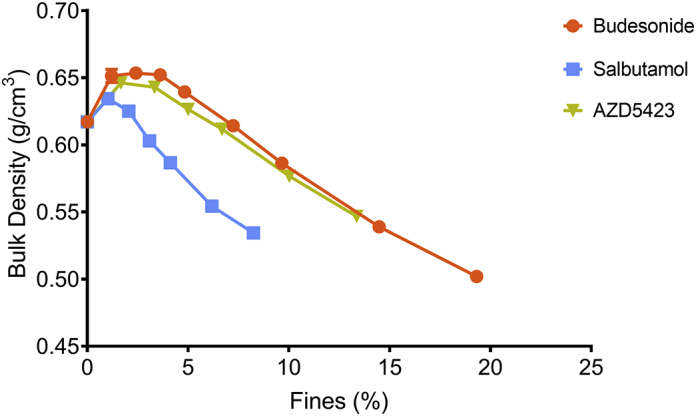
Effect of the proportion (percentage) of fines on the unsettled bulk density of the powders. Average values (*n* = 3) with standard deviation (too low to be visible).

The addition of a small amount of fine material to the carrier increased the bulk density of the mixture compared to the bulk density of the pure carrier powder (0% added fines) for all fine materials (Fig. 5). A further increase in concentration of fines gave a reduction in mixture bulk density for all fine materials, i.e. a maximum was generally obtained in the fines concentration - bulk density profiles of the powders. Both the absolute increase in bulk density and the width of the region before the decrease in bulk density was obtained varied between the APIs. Regarding the decline region of the fines concentration - bulk density profiles, the steepest slope was obtained for salbutamol while similar slopes were obtained for the other two APIs.

**Fig. 6:**
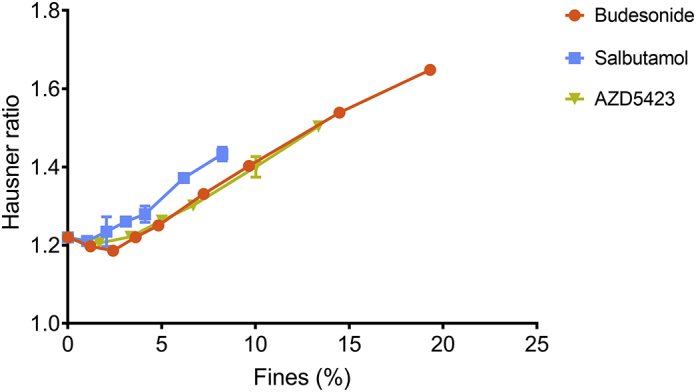
Effect of the proportion (percentage) of fines on the Hausner ratio of the powders. Average values (*n* = 3) with standard deviation (some too low to be visible).

For the fines concentration - Hausner ratio relationships (Fig. 6), a decrease in the Hausner ratio was generally obtained after the addition of a small amount of fines. Thereafter, the Hausner ratio increased. Budesonide and AZD5423 showed almost overlapping profiles while for salbutamol, the Hausner ratio started to increase at a lower concentration of fines, i.e. the salbutamol profile was shifted along the fines concentration axis compared the other APIs.

### 3.4 Dispersibility

**Fig. 7:**
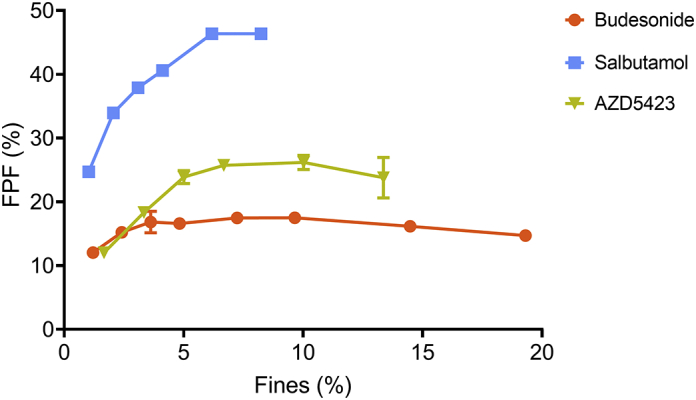
Effect of the proportion (percentage) of fines on the fine particle fraction of the API mixtures. Average values (*n* = 3) with standard deviation.

**Fig. 8:**
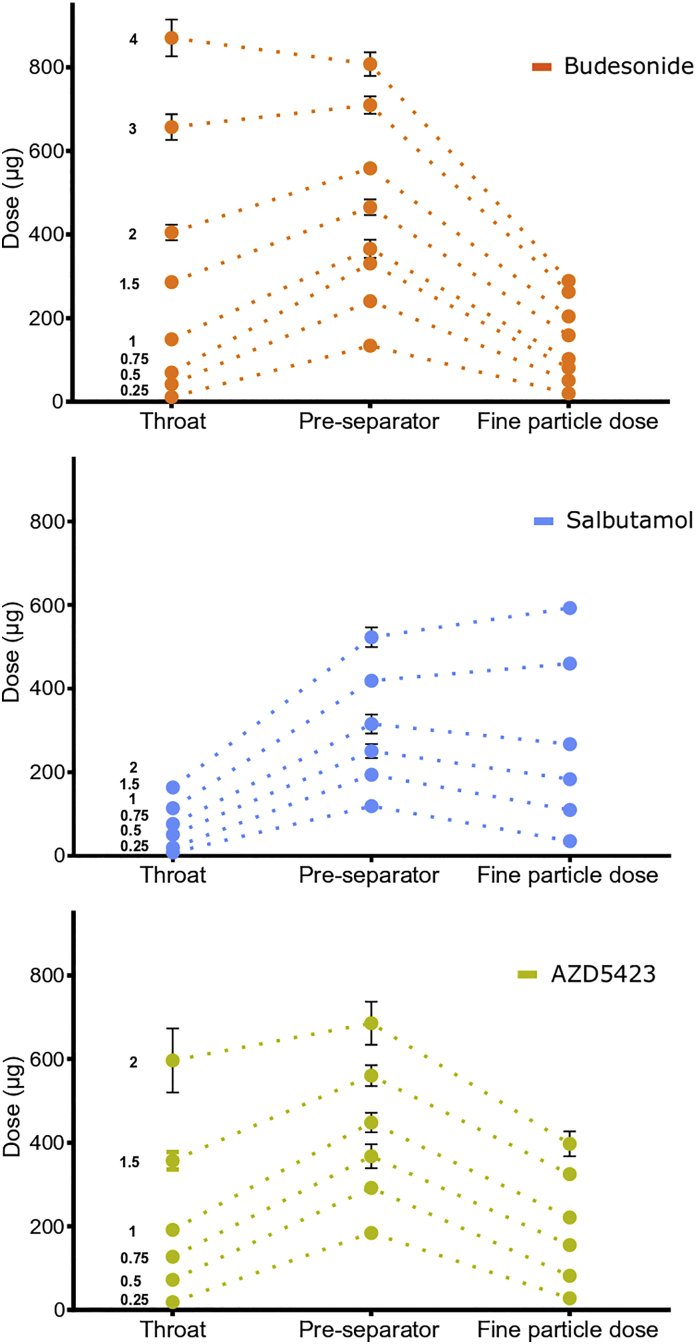
Deposition pattern of all API mixtures (fine particle dose = filter stage). Average values (*n* = 3) with standard deviation.

The dispersibility of the formulations from the FSI setup was assessed by the fine particle fraction (Fig. 7) and the deposition pattern (the distribution of doses at the three dose collection stages) (Fig. 8). For all fine materials, the FPF increased with increasing fines concentration up to a point where the FPF levelled out and reached a plateau after which the FPF decreased slightly. Although the overall trend was similar for all API mixtures, the fines concentration – FPF profiles differed depending on the API regarding both the attained FPF maximum and the concentration of fines at the start of the plateau. For budesonide mixtures, the FPF was generally low (a maximum of about 16%) and a plateau was reached at a fines concentration of about 4%. The AZD5423 mixtures had a higher maximum of about 25% FPF, which was reached at a fines concentration of about 7%. The salbutamol mixtures gave significantly higher FPF values, i.e. a maximum of 46% at a fines concentration of about 7%.

An increased concentration of API in the mixtures, represented by the series of SCR, generally gave increased doses of API deposited at the different stages (Fig. 8). However, the deposition pattern, i.e. the distribution between the stages, differed dependent on the API. At low SCR, deposition in the pre-separator was higher than deposition at the other two stages. For budesonide, an increased SCR gave a larger dose of API deposited in the USP throat while a lower relative dose was deposited in the filter stage. For salbutamol, a low deposition in the throat was generally obtained and the relative dose deposited at the filter stage increased with increased SCR. For AZD5423, an increased SCR gave an almost parallel increase in dose deposited at the respective stage except at the highest SCR, i.e. SCR 2, where the deposition in the throat increased.

## 4 Discussion

In an earlier paper (Rudén et al. 2018), we introduced a blend state concept as a means to describe the different states an adhesive mixture may undergo with the addition of increasing proportions of fines. In that study, one type of carrier particles and one type of micro-particles in different proportions were used. In a follow-up paper (Rudén et al. 2019), the blend state map concept was used to describe and compare the blend properties of different combinations of micro-particles and carriers. In that paper, the importance of the shape and size of the carrier particles for the evolution of the blend state was investigated, again using one type of fines, i.e. micro-particles of lactose. In the investigation presented here, binary adhesive mixtures of one single carrier and micro-particles of three active pharmaceutical ingredients (API) were studied. The objectives were to study similarities and differences in the evolution in blend state between the APIs and to study the relationship between blend state and dispersibility of the mixtures.

### 4.1 Blend state maps

Based on the combination of images of the adhesive mixtures and their packing and flow properties, blend state maps were constructed for all three APIs. In the two earlier papers, we used the surface coverage ratio (SCR) in the presentation of blend state maps. Due to the high inner surface area of AZD5423 particles (Table 1), i.e. the surface area of the open nano-pores, the BET surface areas could not be used as estimates of the enveloped particle surface areas. Instead, permeametry surface areas were used in the calculation of SCR. Particles of AZD5423 had a considerably lower permeametry surface area than the other APIs, which is not a consistent with the low particle diameter. The low permeametry surface area may be explained by a less uniform pore structure of the powder bed AZD5423 held in the sample cylinder of the Blaine apparatus which indicates that the micro-particles of AZD5423 formed agglomerates that remained intact during the compression of the powder in the sample cylinder. An alternative approach to derive estimates of the enveloped surface area is to calculate the surface area from the particle diameter distributions and indications of the particle shape. The latter is difficult to define based on imaging and thus, such values were not calculated. Thus, qualified data of the enveloped surface areas could not be derived for all three APIs and the fines concentration is hence used in all the graphs including the blend state maps in this paper. In addition, the dispersibility of the adhesive mixtures is typically discussed in the literature in relationship to the fines concentration of the blend. However, in order to make a comparison with the evolution of blend states with SCR reported earlier, the corresponding SCR:s (calculated using permeametry surface areas) to the series of fines concentration used are reported in Table 2.

**Fig. 9:**
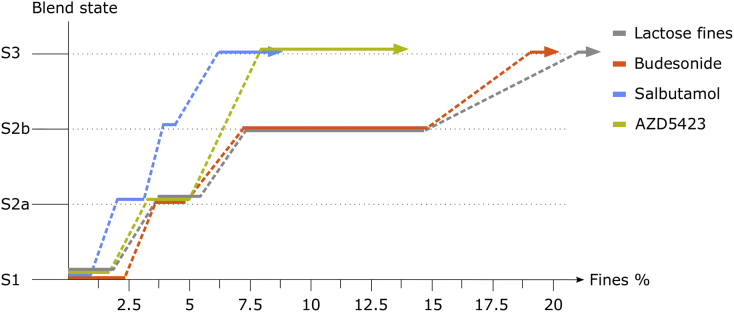
Blend state maps of the mixtures, including lactose fines.

With an increased proportion of fines, the evolution in blend state (Fig. 9) followed for all APIs a step-wise pattern but with different extensions of the stages (the dotted lines indicate the transitions from a lower to a higher blend state). In comparison, the blend state map for lactose fines is also given in the figure (data from Rudén et al. (2018)).

The first blend state (S1), which is associated with an improved flowability and increased bulk density, was clearly distinguished by the powder mechanics measurements (Fig. 5, Fig. 6). The next state (S2a) was reached when flowability and bulk density of the powders started to decrease after the peak values. The subdivision of state 2 into S2a and S2b was based on visual estimation of the structure of the adhesion layer from SEM images. The final state (S3) was determined primarily by visual observations of the presence of large self-agglomerates of fines in the blends (Benassi et al. 2019; Rudén et al. 2019). Formation of self-agglomerates may also lead to a rate change in the bulk density – fines concentration profile, as discussed earlier for the lactose fines (Rudén et al. 2019). The construction of a blend state map is thus somewhat arbitrary but the map nevertheless reflects the evolution of the physical state of an adhesive mixture.

There was a similarity in blend state evolution for the adhesive mixtures independent of the nature of the fines used. However, there was also a clear variation in the transitions of the blend state maps between the APIs although they had similar median particle diameter.

For *Budesonide* the width of each blend state was larger and the transitions from one stage to another thus occurred at higher concentrations of fines compared to salbutamol and AZD5423. Consequently, the concentration of fines at which self-agglomerates appeared (S3) was considerably higher for budesonide indicating a potentially higher drug loading capacity. In addition, the S1 interval was wider, meaning that a larger quantity of fines could be fitted into the cavities of the carrier. As an indication of the volume of open cavities at the carrier surface available for adsorption of fines, a specific pore volume (SPV) can be calculated, as reported earlier (Rudén et al. 2019). The calculation is based on the assumption that at maximum (peak) bulk density, the pores are completely filled with fines while the adhesion of fines to the enveloped carrier surface is negligible. The SPV thus represents a theoretical volume of accessible pores for a specific combination of carrier and fines. In this study, budesonide had the SPV of 0.23 (Table 4), compared to 0.13 and 0.14 for salbutamol and AZD5423 respectively. This indicates that for budesonide, the primary particles could be packed with a higher packing density into the open pores of the carrier during mixing, i.e. budesonide powder seemed more compressible while subjected to the type of forces involved during the mixing (Grasmeijer et al. 2014; Kaialy 2016).

**Table 4:** Specific pore volume of carrier at maximum bulk density of the mixture (i.e. at stage 1) for the respective API.

API (SCR)	Carrier amount (cm^3^)	Fines amount (cm^3^)	SPV
Salbutamol (0.25)	96.1	4.99	0.13
Budesonide (0.5)	91.4	8.58	0.23
Lactose fines (0.25)	95.3	4.70	0.12
AZD5423 (0.25)	94.5	5.44	0.14

After the peak in the bulk density-fines concentration profile, the bulk density gradually decreased (Fig. 5), corresponding to the formation of an adhesion layer on the enveloped surface of the carrier of a gradually increased thickness, i.e. the enveloped volume of the adhesive units increased. Based on the appearance of the adhesive units (Fig. 3), it is concluded that budesonide formed a relatively smooth and homogenous state 2a adhesion layer. With increasing fines concentration, smaller loosely attached agglomerates could be observed on the carrier surfaces corresponding to the transition into stage 2b (at an SCR of about 1.5), Before the transition into stage 3, the carrier surface seemed almost completely covered with fines, which indicates a good adhesion of budesonide to the carriers. Finally, one may note from the light-microscope images that self-agglomerates of budesonide appeared more porous than for the other APIs.

Indeed, for *salbutamol*, all four blend states could be identified but the width of each stage was lower than for budesonide. Salbutamol had among the three APIs the lowest upper concentration level for S1, generating the lowest SPV (Table 4), and the lowest concentration at which self-agglomerates were formed (S3). It is previously reported that nearly spherical spray-dried salbutamol had a higher tendency to pack into open pores of a carrier (Mönckedieck et al. 2017) and a lower tendency to attach to the outer surface of the carriers (Pinto et al. 2018) than more irregular milled salbutamol particles. Thus, the low amount of particles adsorbed into open surface pores of the carrier may, at least partly, be explained by the needle-like shape of the salbutamol particles making packing into open pores of the carrier difficult. In S2, the salbutamol particles initially appeared to adhere to the carrier surface as single particles or as small agglomerates with only a few particles. However, with increased concentration of fines, the bulk density of the mixtures reduced at a faster rate compared to the other two APIs. This means that the carrier particles were distanced from each other to a comparatively high degree due to a growing adhesion layer with high porosity (Rudén et al. 2018). This was supported by the 10.13039/100014281SEM images (Fig. 3), which showed an adhesion layer consisting of small agglomerates distributed over the carrier surface rather than a homogenous coherent layer.

It is reported in the literature that salbutamol is prone to be electrostatically charged during handling which may affect the performance of the mixture (Jetzer and Morrical 2019; Zellnitz et al. 2019). We also observed charging of particles during preparation and handling of the salbutamol mixtures, in contrast to mixtures of the other APIs, which may have affected the evolution of the blend state. As previously mentioned (section 2.6.1), all mixtures were left to equilibrate post mixing for at least one day before characterization to reduce the influence of electrostatic charges on the results.

For *AZD5423,* the width of S1 was in-between budesonide and salbutamol, giving a SPV close to salbutamol but between the other two APIs (Table 4). Also the width of S2a was in-between the two other APIs and a similar blend state map as for lactose fines was obtained for the states 1 and 2a. The decline region of the bulk density fines concentration relationship was for AZ5423 similar to budesonide and nearly overlapping Hausner ratio - fines concentration relationships were obtained for these APIs. Thus, the packing and flow properties of mixtures of these APIs were similar which indicate a similar structural build-up of the adhesion layer. However, if one compare SEM images representing S3 for budesonide and AZD5423 (Fig. 3), the former had a complete, nearly continuous layer of fines of the carrier surface while the latter had an adhesion layer consisting of small agglomerates of fines spread out on the carrier surface.

In summary, adhesive mixtures of all APIs used in this study followed the three main states (S1-3) for structural evolution of adhesive mixtures with increasing concentration of fines. The three key aspects of such a model are the adsorption of fines into surface cavities, the formation of an adhesion layer located at the outer carrier surface and the formation of self-agglomerates of fines. The blend state maps captured differences between the APIs in terms of the width of the states and hence the fines concentration at which the state transitions occurred. The blend state model is thus a potentially useful tool to map mixing properties of an API in relationship to a specific carrier. Based on visual examination of the blends and using powder mechanical properties of the mixture it was concluded that the structure of the adhesion layer in S2 differed between the APIs. Budesonide gave a relatively uniform and coherent adhesion layer on the carrier surface while salbutamol gave a thick uneven layer of low density characterized by small agglomerates dispersed on the carrier surface. The strong effect of salbutamol fines on the bulk density of the adhesive mixture indicates that the agglomerates attached to the surface had a relatively high strength or integrity. AZD5423 developed an adhesion layer thickness similar to budesonide but the layer was more heterogeneous in structure with patches of agglomerated fines attached to the carrier instead of a more continuous layer of fines. Moreover, AZD5423 was more prone to self-agglomerate and formed a S3 at a much lower fines concentration. In the following section, the question of the importance of blend state and adhesion layer structure for the dispersibility of the adhesive mixtures will be addressed.

### 4.2 Blend state – Blend dispersibility relationships

A Fast Screening Impactor (FSI) with three impaction stages (throat, pre-separator and filter) equipped with a ScreenHaler (Thalberg et al. 2016), was used to assess the dispersibility of the adhesive mixtures. The filter stage of the FSI has an aerodynamic cut-off of 5 μm at the flow rate used (60 L/min) and particles collected from this stage are reported as fine particle fraction (FPF) and fine particle dose (FPD).

**Fig. 10:**
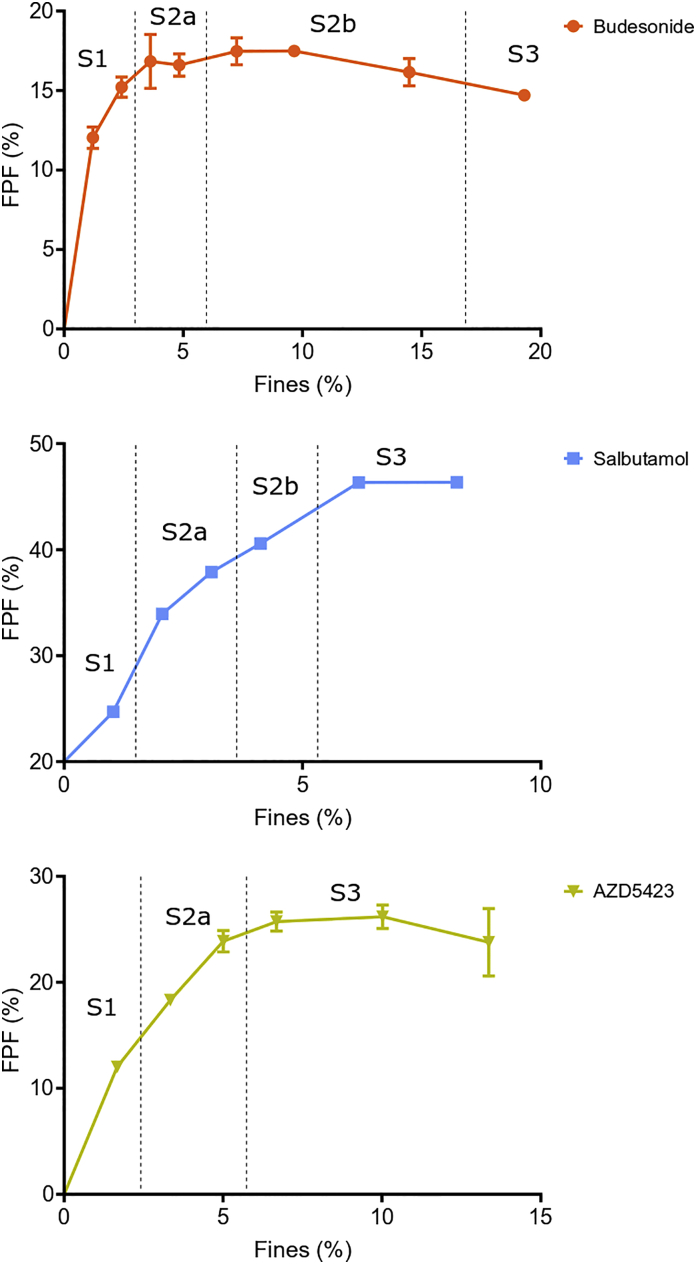
Effect of the proportion (percentage) of fines on the fine particle fraction of the API mixtures combined with the blend state of each API. The transition is set in between blend states. The exact transition point is unknown (see Fig. 10).

**Fig. 11:**
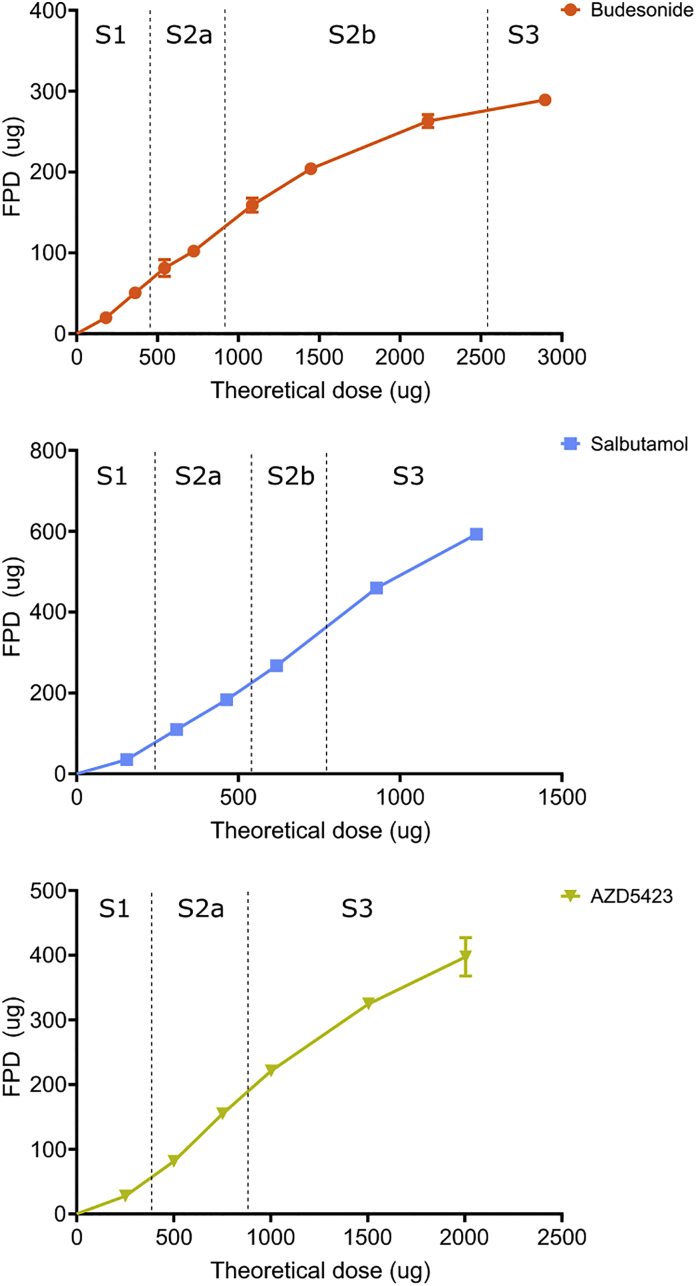
Fine particle dose (FPD) in relation to the theoretical dose combined with the blend state of each API. The transition is set in between blend states. The exact transition point is unknown (see Fig. 10).

In order to examine how the FPF and FPD relate to the blend state, the different blend states for each API were marked out in the relationships between FPF or FPD and fines concentration (Fig. 10, Fig. 11).

Regarding the *fine particle fraction – fines concentration* relationships all three APIs expressed a similar type of bended relationship approaching a constant FPF at the highest fines concentration. For the mixtures categorized as blend state 1, the fraction of fine particles of the emitted dose was generally low but increased substantially and nearly linearly with increased fines concentration obtained. Thus, the S1 part of the relationships was characterized by a relatively low dispersibility, but with a high rate of change in FPF as a function of fines concentration. Thus, it seems that the gradual filling of cavities makes the detachment of fine particles easier. When the mixtures approached and transited into blend state 2, the FPF continued to increase but at a decreasing rate, i.e. the profiles bended clearly. This was especially notable for budesonide, which reached a plateau in the profile already during S2a. For the other APIs, the rate of change ceased when the final blend state (S3) was reached and a plateau in the profiles was obtained. For budesonide and AZ5423, the FPF – fines concentration profile tended to bend downwards in S3.

The type of profile describing the FPF-fines concentration relationship could be divided into three regions, each coupled to the blend state of the adhesive mixtures. In S1 the fine particles are located in open cavities and a low FPF is obtained. The particles are shielded and also relatively strongly adsorbed on the surface inside the cavities and will detach to a limited degree during dose emission. This is consistent with earlier reports in the literature where it is reported that the FPF for particles located in surface cavities are relatively low and will increase when an adhesion layer at the outer carrier surface is formed (De Boer et al. 2005; Hertel et al. 2018; Young et al. 2011). In order to avoid the adsorption of API particles into surface cavities, inert fines have been used to fill such irregularities before admixing of API fines (Grasmeijer et al. 2014; Yeung et al. 2018). When the surface cavities are gradually filled, the particles become less shielded and the detachment will become easier and the FPF increase markedly with fines concentration. In S2 the FPF continues to increase but at a gradually reduced rate and approaches a constant FPF with increased fines concentration, the latter coincides with the transition from S2b or to S3. Finally, S3 is characterized by a nearly constant or even falling FPF. In this state, large self-agglomerates exist which will resist disintegration and dispersion in the air, especially when using an inhaler of a Screenhaler type which is lacking impaction surfaces. Self-agglomerates will thus be emitted from the inhaler and subsequently impact in the throat or the pre-separator (Hertel et al. 2018; Yeung et al. 2019; Young et al. 2011). The formation of a gradually thicker and more uneven adhesion layer in S2 thus facilitates detachment of fines. However, at the beginning of S3 an adhesion layer of unaltered structure has been formed and further addition of fines will increase the concentration of free self-agglomerates which are difficult to disintegrate and FPF will remain constant.

Although the type of FPF-fines concentration relationships and their dependency of the blend state were similar between the APIs, the absolute levels of FPF differed markedly between the APIs (Fig. 7), where salbutamol had the highest FPF, followed by AZD5423 and finally by budesonide which never reached a fine particle fraction higher than 18% irrespective of the API concentration.

It is earlier reported (Thalberg et al. 2012) that the relationship between the performance of an adhesive inhalation powder, in terms of variation in lung dose to the patient, and the fines concentration can be categorized into three regions, i.e. a dilute system region, an intermediate region and a high drug load region. This categorization into three regions is principally similar to the sub-division of the FPF-fines concentration profiles obtained in this study and here proposed to be controlled by the blend state.

Regarding the *fine particle dose - theoretical dose* relationships, the profiles were weakly sigmoidal (Fig. 11). The initial positively bended part occurred predominantly during blend state 1. During S2, the relationships were nearly linear and in the late part of S2 (budesonide) or in S3 (salbutamol and AZD5423) the profiles began to bend negatively. The type of profile describing the FPD-theoretical dose relationship can be coupled to the blend state of the adhesive mixtures in the same way as for the FPF-fines concentration relationships. Also here, the absolute levels of FPF differed between the APIs in the same way as for the FPF, i.e. salbutamol had the highest FPD, followed by AZD5423 and, finally by budesonide.

It is concluded that the dispersibility of APIs in an adhesive mixture can be linked to the blend state. However, the type of API affects the dispersion degree in the different blend states in terms of absolute level of the FPF and FPD. The difference between the APIs are especially notable in S2, the state corresponding to the formation and gradual build-up of the adhesion layer at the enveloped carrier surface. Thus, the structure of the enveloped adhesion layer is an important factor explaining the difference in dispersion degree between the APIs which depends on the nature of the API.

*Budesonide* formed, in relative terms, a dense, coherent adhesion layer of high weight before S3 was entered. Detachment of API particles, especially as single particles, from such a layer may occur to a low extent and the FPF and FPD will be low. In addition, the API can possibly detach as small flakes or aggregates from the surface. The stage distribution in the impactor of budesonide (Fig. 8) shows that a major portion of emitted API was deposited in the throat or in the pre-separator and with increased SCR, the fraction of API deposited in the throat increased. This indicates that relatively strong flakes or aggregates were detached rather than single particles and that the aggregates resisted dispersion in the air stream.

*Salbutamol* generated much higher fine particle fractions than budesonide throughout the investigated range of fines concentration. Salbutamol developed a relatively thick and porous adhesion layer. It is proposed that this layer consisted of small agglomerates of salbutamol and if they do not develop strong interactions between themselves, the agglomerates may be relatively easy to detach and subsequently disperse in the air stream, thus giving high FPF and FPD. The effect of SCR on the stage distribution in the impactor of salbutamol was clearly different to budesonide (Fig. 8). At the lowest SCR, i.e. at S1 and beginning of S2, the stage distributions between budesonide and salbutamol were similar. With increased SCR, i.e. at fines concentrations where the heterogeneous adhesion layer was formed, the stage distribution differed markedly and for salbutamol, an increased fraction of API impacted at the fines stage.

For *AZD5423*, the FPF – fines concentration relationship coincided at low fines concentration with the budesonide profile. It is argued above that the adhesion layer formed in S2 consisted of particles heterogeneously spread out as dense patches on the enveloped carrier surface, patches from which single particles may be relatively hard to detach. At some point of fines concentration, larger agglomerates that were easier to detach were formed and the FPF increased. However, compared to salbutamol, the agglomerates seemed more difficult the completely disintegrate and the impactor stage distribution was similar to the distribution for budesonide.

**Fig. 12:**
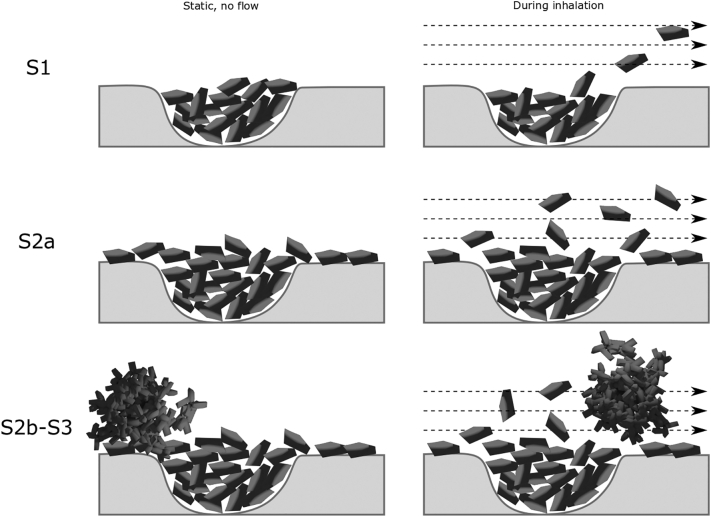
Proposed detachment mechanisms dependent on the blend state of the adhesive mixture.

An illustration of the different mechanisms of aerosolization during inhalation is presented in Fig. 12. These mechanisms are also earlier discussed in the literature (Hertel et al. 2018; Yeung et al. 2018; Young et al. 2011). In the first state S1, the fine particles are released from the cavity as single particles. In the S2a state, the fines are also predominantly released as single particles but since they are more exposed to the air they detach easier (Young et al. 2011). In the agglomerated states S2b and S3, a combination of single particles and self-agglomerates of fines will detach from the carrier surfaces (Thalberg et al. 2016).

It is concluded that the structure of the adhesion layer is an important factor explaining the relationship between blend state and blend dispersibility. It is reasonable that the nature of the API is critical for the development of the adhesion layer structure. Regarding the APIs used in this study, the particle size was similar but there were differences in particle shape. Particle shape may thus be an important API property explaining the observed differences in blend dispersibility, i.e. an elongated particle shape tends to form a more porous and heterogeneous adhesion layer which facilitated the detachment and dispersion processes during aerosolization (Adi et al. 2008; Mönckedieck et al. 2017). However, other API properties may also be important, such as the surface energy affecting the strength of the adhesive and cohesive interactions in the blend.

In this study, there was no mouthpiece connected to the Screenhaler to facilitate dispersion of agglomerates. The intention by using a relatively mild treatment of the mixtures during aerosolization was to improve the discrimination of the inherent dispersibility of the different mixtures. The dispersion capability is known to vary between inhalers (Donovan et al. 2012; Grasmeijer et al. 2013) and the use of a mouth piece fitted to the ScreenHaler will probably improve the degree of dispersion of the mixtures (de Boer et al. 2012; Donovan et al. 2012; Weers and Miller 2015). Initial experiments in our laboratory indicates that the degree of dispersion will increase also of the adhesive mixtures used in this study if a Turbuhaler® mouth piece is connected to the ScreenHaler.

The mixing procedure used in this study was the same as in our previous investigations (Rudén et al. 2018; Rudén et al. 2019) and it is assumed that under these mixing conditions the mixtures reached equilibrium or end-point blend states. It is however reasonable that the relationships between blend state and proportion of fines will be different if other mixing conditions are used, affecting also the blend state – blend performance relationships. It is for example reported (Grasmeijer et al. 2013; Hertel et al. 2017; Thalberg et al. 2020) that the mixing time and intensity affects the detachment process of fine particles from the carrier, indicating a mixture-condition dependency of the properties of the adhesion layer. Thus, the importance of mixing conditions for the formation and properties of the adhesion layer deserves to be further explored.

### 4.3 Relationships between powder mechanics and dispersibility performance

**Fig. 13:**
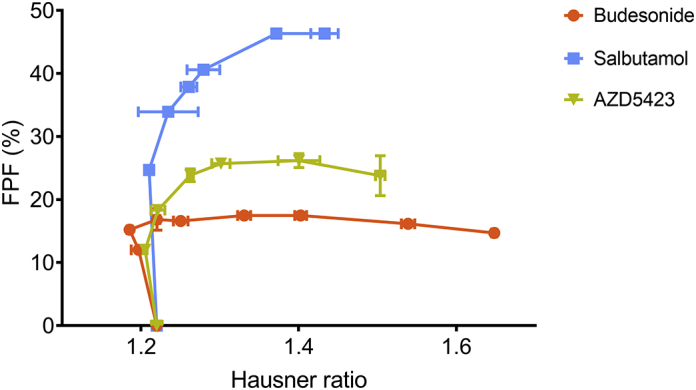
Relationships between Hausner ratio and fine particle fraction. Average values (*n* = 3) with standard deviation.

Impactor testing is crucial in the development of inhalation powders in order to demonstrate reproducibility and stability of DPI formulations. Since such experiments are tedious, the prediction of the dispersibility based on the measurement of other powder properties using faster experimental procedures is of interest, such as the measurement of powder mechanical properties. Several studies are reported in the literature with the aim of using powder mechanical methods as prediction tools for the dispersibility of adhesive mixtures (Hertel et al. 2018; Jones et al. 2010; Kinnunen et al. 2014; Sun et al. 2020). Hertel et al. (2018) studied the influence of fines concentration on the powder mechanics of formulations based of two different APIs and two differently sized carriers. Using two different inhalers, the dispersibility performance of the formulations were assessed and the FPF could be related to events observed in the powder mechanics measurements (fluidization energy, permeability, aeration ratio). Similar attempts and relationships were found by Sun et al. (2020) with an array of different powder mechanical techniques and using principal component analysis. Kinnunen et al. (2014) measured the basic flowability energy and fluidization energy and found no clear correlation with the observed FPF of the formulations. Jones et al. (2010) measured the angle of repose of binary and ternary adhesive mixtures and they could not find any correlation to the fine particle fraction. However, Jones et al. concluded that freely flowable powders appeared to disperse poorly. In this study, we did not use the same type of powder mechanical techniques as in the previously mentioned studies, but according to our first study (Rudén et al. 2018) and the study by Kinnunen et al. (2014), different powder mechanical techniques provide similar relationships and can thus be interchangeable. Coinciding relationships between the FPF and the mixture flowability were not obtained for the three API mixtures (Fig. 13). One may however note that at a Hausner ratio of around 1.4 the maximal FPF was reached and did not further change with decreasing flowability.

## 5 Conclusion

In this paper, we have constructed blend state maps for binary carrier-based adhesive mixtures using a single type of carrier and three drug micro-particles and thereafter studied the relationship between the blend state and the blend dispersibility. Based on the findings we conclude that:

• The adhesive mixtures of all drugs followed the earlier proposed states (S1-3) for structural evolution of adhesive mixtures with increasing concentration of drug micro-particles, supporting that the blend state model is a general concept describing adhesive mixtures.
• The width of the different states, and hence the drug concentration at which the state transitions occurred, differed between the drugs and the blend state model is thus a potentially useful tool to map mixing properties of a drug to be used in adhesive mixtures.
• The structure of the adhesion layer in state 2 differed between the drugs, from a relatively uniform and coherent adhesion layer to an uneven layer with small drug agglomerates dispersed on the carrier surface.
• Both the fine particle fraction-fines concentration relationship and fine particle dose-theoretical dose relationship could be divided into three regions and each region was coupled to the blend state of the adhesive mixtures.
• The dispersion degree in the different blend states, i.e. absolute levels of fine particle fraction or fine particle dose, differed between the drugs and the difference was especially notable in state 2. Thus, the structure of the enveloped adhesion layer is important for the layer dispersibility. However, the question of the impact of inhalers with different dispersion capability on the adhesion layer structure-blend dispersibility relationship remains to be investigated.
• The geometrical shape of the drug micro-particles is critical for the development of the adhesion layer structure.
• The flowability of the adhesive mixtures could not be generally correlated to their dispersibility and the predictive potential of mixture flowability for mixture dispersibility is hence questionable.

## Declaration of Competing Interest

The authors declare that they have no known competing financial interests or personal relationships that could have appeared to influence the work reported in this paper.
